# On the optimality of the enzyme–substrate relationship in bacteria

**DOI:** 10.1371/journal.pbio.3001416

**Published:** 2021-10-26

**Authors:** Hugo Dourado, Matteo Mori, Terence Hwa, Martin J. Lercher

**Affiliations:** 1 Institute for Computer Science and Department of Biology, Heinrich Heine University, Düsseldorf, Germany; 2 Department of Physics, University of California at San Diego, La Jolla, California, United States of America; Duke University, UNITED STATES

## Abstract

Much recent progress has been made to understand the impact of proteome allocation on bacterial growth; much less is known about the relationship between the abundances of the enzymes and their substrates, which jointly determine metabolic fluxes. Here, we report a correlation between the concentrations of enzymes and their substrates in *Escherichia coli*. We suggest this relationship to be a consequence of optimal resource allocation, subject to an overall constraint on the biomass density: For a cellular reaction network composed of effectively irreversible reactions, maximal reaction flux is achieved when the dry mass allocated to each substrate is equal to the dry mass of the unsaturated (or “free”) enzymes waiting to consume it. Calculations based on this optimality principle successfully predict the quantitative relationship between the observed enzyme and metabolite abundances, parameterized only by molecular masses and enzyme–substrate dissociation constants (*K*_m_). The corresponding organizing principle provides a fundamental rationale for cellular investment into different types of molecules, which may aid in the design of more efficient synthetic cellular systems.

## Abstract 2

This study shows that in E. coli, the cellular mass of each metabolite approximately equals the combined mass of the free enzymes waiting to consume it; this simple relationship arises from the optimal utilization of cellular dry mass, and quantitatively describes available experimental data.

## Introduction

Bacterial growth relies on the organized activity of thousands of chemical reactions. Regulation of enzyme abundances and activities finely tunes the corresponding fluxes to match cellular needs [1]. The regulation of protein expression is subject to constraints such as limited ribosomal capacity [2], constant density of macromolecules or dry mass [3–5], and membrane surface area [6]. Each of these constraints can be physiologically relevant in specific conditions, and, in each case, the constraint limits the protein mass that can be produced or allocated in the cell [2].

However, the fluxes of intracellular reactions depend not only on enzyme expression, but also on substrate concentrations. As fluxes need to be balanced in steady-state growth, this dependence leads to mechanistic constraints between enzyme and substrate levels. Systems biology has only recently started to explore the consequences of these relationships on the organization of metabolic systems and on regulatory strategies, such as feedback inhibition, at the genome-scale level [7–10]. The interdependence of fluxes *v* and the concentrations of enzymes [*E*] and metabolites [*S*] are illustrated by the simplest example of enzyme-limited kinetics, the Michaelis–Menten rate equation

$$v=k_{cat}\cdot [E]\cdot \frac{\left[S\right]}{[S]+K_{m}}$$ (1)

**Fig 1:**
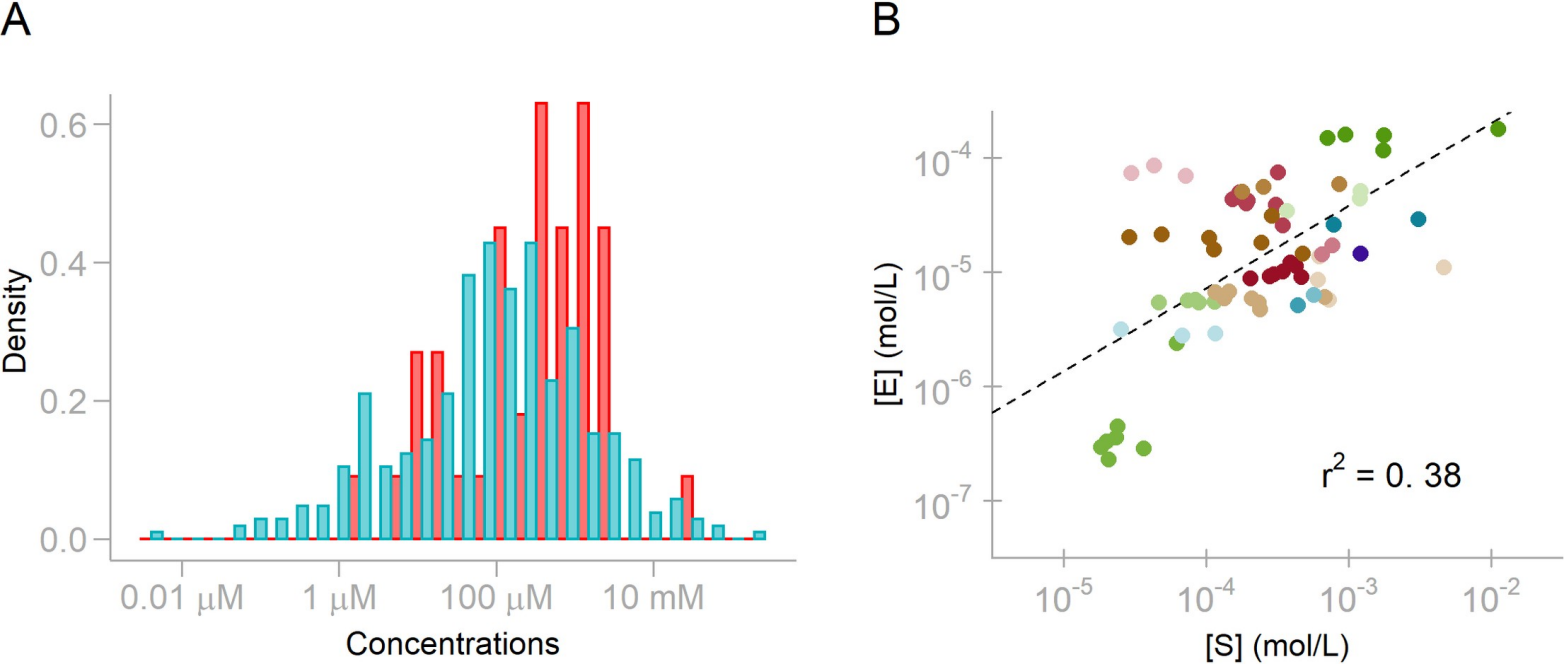
Dissociation constants *K*_*m*_ provide a natural scale for the relationship between substrate and enzyme concentrations. **(A)** Log-scale histograms of observed metabolite concentrations [*S*] (red) [12] and the geometric means of corresponding *K*_m_ values (blue) [11]. **(B)** Correlation between the molar concentrations of enzymes and their substrates. The underlying data can be found in S1 Data.

Here, *k*_cat_ is the turnover number, and the kinetic interaction of substrates with their consuming enzymes is parameterized by *K*_m_, the enzyme–substrate dissociation (or Michaelis) constant. *K*_m_ has the unit of concentration and hence provides a natural scale for the substrate abundance, [*S*]. Typical *K*_m_ values for cellular reactions are in the range of 10 μM to 1 mM (median 98 μM; cyan bars, **Fig 1A**) [11]. Metabolomic measurements in glucose minimal medium found the concentrations of the most abundant metabolites to be of similar magnitude (red bars, **Fig 1A**) [12], with concentrations typically 2 times larger than the corresponding *K*_m_ (**Fig 1A, Fig A in S1 File**). Thus, the enzyme saturation factor [*S*]/([*S*]+*K*_m_) is typically around two-thirds, implying that even for enzyme species actively involved in biosynthesis, one-third of the proteins make no contribution to metabolic fluxes at each point in time. Accordingly, substrate availability is an important factor limiting cellular efficiency and hence fitness [13].

It is commonly assumed that in vivo metabolite concentrations are a consequence of the biochemical properties of each metabolite and of the enzymes by which it is consumed [9,11,14]. However, if cellular efficiency is indeed limited through idle, unsaturated enzyme fractions, it is conceivable that natural selection would favor higher saturation for more highly expressed enzymes, whose idle fractions occupy more cellular resources. To explore this possibility, we collected data on the concentrations of substrates and the dominant enzymes consuming them based on published studies on *Escherichia coli* [12,15]; here, “dominant” refers to the enzyme with the highest proteome fraction compared to all others competing for the same substrate (Materials and methods, “Concentrations” and “Dominant enzymes”). The molar concentrations of *E*. *coli* proteins and their substrates are indeed correlated (**Fig 1B**; Pearson *r*^2^ = 0.39, *P* = 2.2 × 10^−8^): 39% of the variability in substrate concentrations can be predicted from the concentrations of the corresponding dominant enzymes. In the following, we show how a simple, quantitative description of this observation can be derived as an optimality principle that combines enzyme kinetics with a constraint on resource allocation.

## Mechanistic link between enzymes and substrates

To analyze the interdependence of enzyme and substrate abundances, we first focus on the simple case of Michaelis–Menten kinetics, Eq (1). Only a fraction of enzymes is bound to the substrate and catalyzes the reaction, while the remainder, of concentration [*E*_free_], does not directly contribute to the reaction flux. We can rewrite the Michaelis–Menten Eq (1) to highlight this “inefficiency” as

$$v=k_{cat}\cdot (\left[E]-[E_{\mathrm{free}}\right]),$$ (2)

where the concentration of free enzymes is a function of total enzyme and substrate concentrations

$$[E_{\mathrm{free}}]=\frac{\left[E\right]}{1+\left[S\right]/K_{m}}$$ (3)

For efficient enzyme usage, the fraction of free enzymes should be as small as possible, [*E*_free_]≪[*E*]. However, to achieve this, substrate concentrations must be kept much above *K*_m_. Eq (3) and its generalizations thus exhibit a general trade-off faced by living cells: For a given reaction flux, low substrate concentrations lead to inefficient enzyme utilization, while efficient enzyme allocation requires high substrate concentrations.

To assess the relevance of this trade-off, we looked at data from a recent quantitative metabolomics experiment for *E*. *coli* grown on glucose minimal media [12], which observed a total dry mass fraction of 3.1% for 43 assayed metabolites, mostly from central carbon metabolism. The dry mass fraction of cytosolic proteins that are capable of consuming these metabolites is 15.3% (Materials and methods, “Concentrations”). If roughly 70% of these enzymes are bound to substrates (*S/K*_*m*_~2.3, **Fig 1A**), the remaining free enzymes would account for 4.6% of dry mass, making the dry mass contributions of the assayed metabolites and of the corresponding free enzymes comparable. Intuitively, inefficiencies of a few percent may seem low. However, population genetical models show that a relative fitness difference of *s* between members of a population leads to extinction of the less fit strain unless |s|<<1/*N*_e_ (with *N*_e_ the effective population size) [16]; with typical effective population sizes of *N*_e_≈10^8^ in natural bacterial populations [17], a strain that could avoid wasting even 0.1% of its resources would be under substantial positive selection.

The total cell density (its mass per volume) is the sum of its aqueous density and its dry weight per volume (dry mass density); the fraction of dry mass in the total density is approximately constant, at 30% across growth conditions [18,19]. The optimal allocation of the protein part of this mass in schematic whole-cell models has provided qualitative explanations for several experimental observations in *E*. *coli*, such as the approximately linear scaling of the ribosomal protein fraction with growth rate [20–25], optimal and suboptimal regulatory strategies [24–26], and the emergence of overflow metabolism with increasing nutrient quality [20,27–29].

While these studies considered only the protein part of the dry mass density, a given flux through an enzymatic reaction is determined by the concentrations of both the enzyme and the metabolites involved. Metabolites also influence the diffusion and the free energy of other molecules; they hence contribute to molecular crowding, despite being smaller than proteins and accounting for a smaller fraction of the dry weight. The most straightforward way to account for the observed constancy of dry mass density across growth conditions is thus to account for all dry mass components equally. Accordingly, we now explore the consequences of a limited total dry mass density on optimally efficient enzyme–substrate systems; this analysis results in a surprisingly simple quantitative relationship between the contributions of enzymes and their substrates to the dry mass density. This relationship accounts quantitatively for the relationship between the cell’s investment into enzymes and their substrates (**Fig 1B**), as well as for the comparable dry mass fractions of metabolites and the free enzymes waiting to consume them.

## Enzyme–substrate optimality

Let us consider the total contribution of an enzyme *E* (with molar mass *m*_*E*_ and mass density *c*_*E*_ = *m*_*E*_[*E*]) and its substrate *S* (with molar mass *m*_*S*_ and dry mass density *c*_*S*_ = *m*_*S*_[*S*]) to the cellular dry mass density:

$$M_{total}=m_{S}[S]+m_{E}[E]=c_{S}+c_{E}$$ (4)

At constant dry mass contribution *M*_total_, the maximal reaction flux occurs at a unique combination of substrate and enzyme concentrations. For the irreversible Michaelis–Menten kinetics of Eq (1), the optimal contribution of the substrate to dry mass per volume equals the corresponding contribution of the free enzyme molecules waiting to consume it:

$${m_{S}[S]}^{*}=m_{E}{\left[E_{free}\right]}^{*}=\frac{m_{E}{\left[E\right]}^{*}}{1+{\left[S\right]}^{*}/K_{m}}$$ (5A)

or, equivalently,

$$c_{S}^{*}=c_{E,free}^{*}=\frac{c_{E}^{*}}{1+c_{S}^{*}/{\tilde{K}}_{m}},$$ (5B)

where we also scaled the dissociation constant to mass concentrations, ${\tilde{K}}_{m}=m_{S}K_{m}$; here and below, asterisks (*) indicate values optimal for reaction flux.

**Fig 2:**
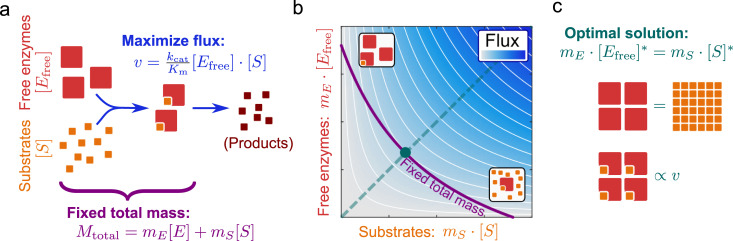
Derivation of the optimal relationship between enzyme and substrate concentrations. **(A)** Irreversible Michaelis–Menten kinetics for enzyme *E* (large red squares) consuming substrate *S* (small orange squares), acting under a constraint on total dry mass for the reaction, *M*_total_. **(B)** Contour plot of the flux dependence on substrate and free enzyme mass concentrations. Blue shading is proportional to flux; white contour lines trace identical flux values at different combinations of substrate and free enzyme concentrations. The magenta line indicates the combined mass concentration of substrate and total enzyme at the limit *M*_total_; maximal flux is achieved on the diagonal (cyan). Equivalently, the diagonal indicates the minimal cellular investment into substrate and free enzyme mass concentration at constant flux *v* (along the corresponding contour line). **(C)** Cartoon illustrating the relationship between enzyme and metabolite concentrations in the optimal solution (the cyan dashed line in (B)). A general mathematical derivation for the optimal relationship between metabolite and enzyme concentrations in reaction networks is provided in Materials and methods (“Derivations”).

The derivation of this relationship is illustrated in **Fig 2** (a formal derivation is given in Materials and methods, “Derivations”). **Fig 2A** illustrates a simple reaction, where enzymes (large red squares) convert metabolites (small orange squares) to products according to irreversible Michaelis–Menten kinetics (Eq (2)). **Fig 2B** shows how the reaction flux *v* (blue shading) scales in proportion to the mass concentrations of free enzymes and substrates. At constant combined mass concentration (density) of enzymes and substrates (violet line), maximal flux is achieved on the diagonal (cyan), where the contributions of free enzymes and substrates are equal (illustrated in **Fig 2C**). From a complementary view point, at this optimal flux value, *M*_total_ represents the minimal possible joint dry mass contribution of enzyme and substrate: This state represents the most parsimonious—or most efficient—dry mass allocation at the given reaction output.

A generalization to reaction networks, with enzymes consuming multiple substrates and substrates consumed by multiple reactions, leads to a very similar equation: Each substrate mass concentration equals the mass concentration sum over all free enzyme species *E*_*i*_ waiting to consume the substrate

$$c_{S}^{*}=\sum_{i}c_{E_{i},free}^{*}=\sum_{i}\frac{c_{E_{i}}^{*}}{1+c_{S}^{*}/{\tilde{K}}_{m,i}}$$ (6)

(Materials and methods, Eq (37)). Further extensions to other irreversible kinetic rate laws (such as metabolite inhibition, Hill kinetics, or stoichiometries other than 1:1) can be derived formally in the same way as Eq (6). Eq (6) and its extensions can be viewed as an approximation to a network-level description of maximal cellular steady-state growth[30], which accounts for the total dry mass conservation while ignoring details of the mass conservation of individual cellular components (**Text A in S1 File**).

**Fig 3:**
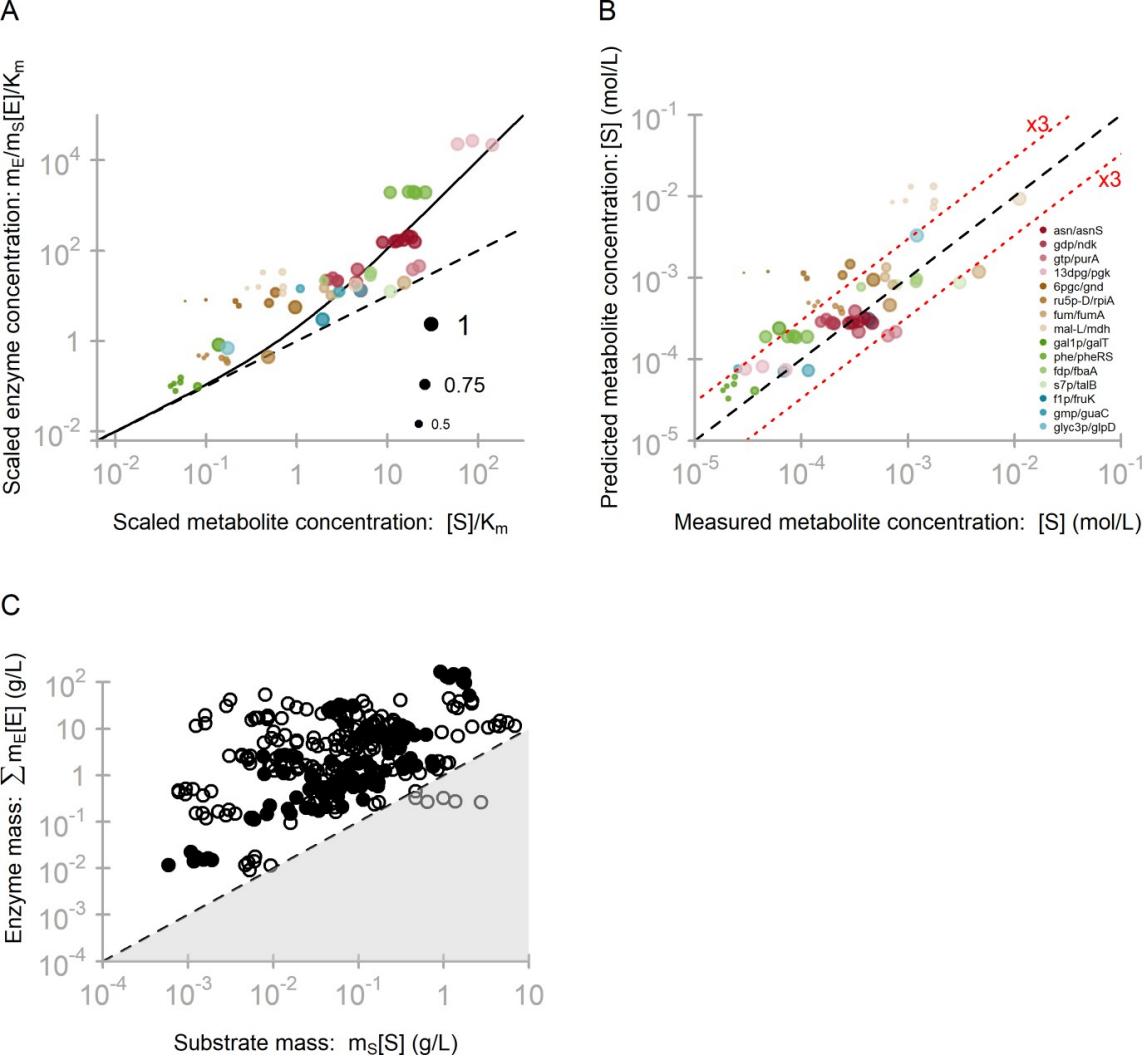
Experimentally observed enzyme [15] and metabolite [12] concentrations reflect the predicted optimal scaling. **(A)** If a single enzyme *E* dominates the total enzyme mass consuming substrate *S* (Materials and methods,”Dominant enzymes”), we can use Eq (5), rewritten for scaled enzyme concentration, $e=c_{E}^{*}/{\tilde{K}}_{m}=m_{E}{\left[E\right]}^{*}/{(m_{S}K}_{m})$ (y-axis), and scaled substrate concentration, $s=c_{S}^{*}{/{\tilde{K}}_{m}=[S]}^{*}/K_{m}$ (x-axis), resulting in the prediction *e* = *s* (1+*s*) (solid line). Data points are color coded by reaction (see abbreviations in (B) and full names in **S2 Data**). Point sizes represent the saturation factor of the enzyme by the substrate, with the highest saturation factor for each enzyme–substrate pair set to 1.0. **(B)** Comparison of experimentally observed (x-axis) and predicted (y-axis) molar metabolite concentrations. Color coding as in panel a. **(C)** As predicted by Eq (6), the combined mass concentration *E*_total_ = $\sum_{i}c_{E_{i}}=\sum_{i}m_{E_{i}}[E_{i}]$ of the enzymes *E*_*i*_ consuming a given substrate *S* is higher than the substrate mass concentration *c*_*S*_ = *m*_*S*_[*S*]. Solid points show substrates for which irreversible enzymes contribute ≥50% to *E*_total_; circles show substrates for which reversible enzymes (some of which may produce rather than consume the metabolite) contribute >50% to *E*_total_. The underlying data can be found in **S2 Data**.

The predictions from Eq (5) become independent of the considered reactions when we scale enzyme and metabolite mass concentrations by ${\tilde{K}}_{m}$, the dissociation constant (in mass units): e* = s*·(1+s*), with $e^{*}≔c_{E}^{*}/{\tilde{K}}_{m}$ and ${s^{*}≔c_{S}^{*}/\tilde{K}}_{m}$. As shown in **Fig 3A**, this predicted relationship (solid line) provides a quantitative description of the observed *E*. *coli* data across several orders of magnitude of enzyme and substrate concentrations [12,15] (*N* = 66, *r*^2^ = 0.57, *P* = 3 × 10^−13^ for predicted versus observed substrate concentrations across minimal media, **Fig 3B**; geometric mean fold error (GMFE) = 2.49).

It is worth emphasizing that the predicted relationship between substrate and enzyme mass concentrations contains no fitting parameters; it is based solely on dissociation constants determined in in vitro experiments [31–33]. It can easily be shown that when predicting substrate concentrations from enzyme concentrations according to Eq (5), uncertainties in the values of dissociation constants lead to relative errors in the substrate concentrations of at most the same magnitude, $\frac{\Delta {\left[S\right]}^{*}}{{\left[S\right]}^{*}}\leq \frac{{\Delta K}_{m}}{K_{m}}$ (Materials and methods, Eq (23)). There is no reason why the experimental estimates of dissociation constants should be biased in support of our predictions. In the absence of Eq (5), there would thus be no reason why the data in **Fig 3A** is distributed around the solid line, just above the plot’s diagonal (which describes equal mass concentrations, $c_{s}^{*}=c_{E}^{*}$), and no reason why the substrate concentrations predicted from enzyme concentrations should be mostly within a factor of 3 of the observed values (**Fig 3B**), a range that is compatible with the combined experimental uncertainty of metabolomics and dissociation constant measurements. This consistency hence constitutes strong a posteriori support for our assumptions.

For **Figs 1** and **3**, we defined “dominant” enzymes as those that constitute at least half of the total protein mass capable of consuming a given metabolite. While this threshold of 50% is to some extent arbitrary, it means, according to Eq (6), that the substrate concentration is mostly determined by this one protein: The combined effect of all other enzymes on the substrate concentration is expected to result in at most a 2-fold error. Choosing alternative cutoffs does not affect the overall conclusions; as expected, the predictions get more accurate at higher cutoffs (**Fig B in S1 File**).

The number of data points in **Fig 3A** is determined by the requirements of Eq (5) (for details, see Materials and methods, “Dominant enzymes”). The most important restriction is that the metabolite’s absolute concentration must have been quantified experimentally in the same strain and condition as the proteome. Moreover, the approximation of Eq (6) with Eq (5) requires that one enzyme dominates the sum in Eq (6), here defined as contributing at least 50% of the total enzyme mass able to consume the metabolite (see also **Fig B in S1 File**).

To include more data points, we can make another approximation to Eq (6) that does not require the existence of a dominant enzyme and is independent of *K*_m_: In the optimal state, each substrate mass concentration must be smaller than the combined mass concentrations of its consuming enzymes, $c_{S}\leq \sum_{i}c_{E_{i}}^{*}$ (i.e., ${m_{S}[S]}^{*}\leq \sum_{i}m_{E_{i}}{\left[E_{i}\right]}^{*}$). While molar concentrations of substrates are much higher than those of enzymes (**Fig 1B**), the substrate mass density appears to provide a lower bound for the corresponding enzyme masses density, as predicted: Almost all data points in **Fig 3C** fall above the diagonal. Reversible enzymes (i) may produce rather than consume the substrate; and (ii) may operate close to thermodynamic equilibrium; we thus expect substrates for which reversible enzymes contribute the majority of the total enzyme mass (open circles in **Fig 3B**) to deviate, on average, more from the lower bound than substrates for which irreversible enzymes dominate (solid dots).

If the dominant enzyme for a given metabolite remains the same across multiple conditions, we expect the corresponding points to follow the prediction line from Eq (5)—with different positions along the x-axis corresponding to differences in the enzyme’s saturation. This effect can be seen for galactose-1-phosphate uridylyltransferase (GalT): GalT is expressed at high levels only in growth on galactose, which is the only condition where it must sustain high fluxes. In other conditions, the enzyme and its substrate alpha-D-galactose 1-phosphate (GAL1P) show a correlated decrease (**Fig 3A**), demonstrating that Eq (5) can also apply at low reaction fluxes.

The predictions do not match the data in **Fig 3A** perfectly. For each enzyme–substrate pair, point sizes reflect the relative saturation; smaller points indicate a lower saturation and hence a higher fraction of free enzymes. The highest saturation for each pair (dot size 1.0 in **Fig 3A**) typically corresponds to the largest reaction flux and is generally associated with a relatively good agreement between data and predictions (*N* = 15, *r*^2^ = 0.72, GMFE = 1.96, **Fig C in S1 File**). Substrate concentrations and hence saturation are often much lower in other conditions (smaller dots in **Fig 3A**). By contrast, the corresponding enzyme concentrations are typically maintained at high levels; a notable exception is GalT, which has a central metabolic function only in growth on galactose, and for which enzyme concentrations are much lower in other conditions. This observation of near-constant enzyme concentrations across conditions indicates a limit to the optimal resource allocation quantified in Eqs (5) and (6): For most enzyme–substrate pairs with similar metabolic roles across multiple conditions, the cellular organization appears to approximate optimal metabolic efficiency at the highest flux condition (where cellular costs for this reaction are highest), but may not reduce enzyme concentrations specifically in conditions that require lower fluxes.

## Conclusions

In this work, we have shown that the experimentally observed enzyme–substrate relationship is roughly consistent with an optimal allocation of cellular mass between catalysts and their substrates, where the cellular mass of a metabolite equals the combined mass of all free enzymes waiting to consume it. For simple, irreversible Michaelis–Menten kinetics (Eq (1)), this relationship follows directly from the proportionality of the reaction flux to the concentrations of substrate and free enzymes and from the assumption of a limited dry mass density (**Fig 2**). If all enzymes consuming a given metabolite make up only a small combined proteome fraction, the optimal relationship causes enzymes to be, on average, only weakly saturated with that metabolite.

How could the cell achieve such an optimal balance between the concentrations of metabolites and enzymes across changing environments? To do so would demand very detailed, environment-dependent regulation of individual protein concentrations. The machinery required for such detailed fine-tuning would likely be very costly and might be less robust to perturbations than a simpler, approximate regulatory strategy. Due to this trade-off, natural selection may have favored the evolution of an approximate, robust implementation of the optimal enzyme–metabolite balance, potentially explaining why enzyme concentrations are roughly constant across conditions (**Fig 3A**). Moreover, a trade-off between enzyme–metabolite optimality and regulatory costs may also be consistent with the observation that protein concentration changes across growth conditions are often regulated not at the level of each individual protein, but at the level of complete pathways or protein sectors [2,21,34,35], controlled by global factors such as Crp [36].

Our derivation of the proposed optimal balance between catalysts and their substrates is based on (i) the assumption of a constant dry mass density, which encompasses all intracellular nonwater molecules regardless of their molecular sizes. Accounting for all dry mass components equally is simply the most straightforward way to account for the observed constancy of dry mass density across growth conditions in *E*. *coli* [18,19]. Previous studies have independently focused on 2 different types of concentration bounds: (ii) a limit on the volume concentrations of large molecules such as proteins, DNA, and RNA, termed “macromolecular crowding” [3,20]; and (iii) a limit on the molar concentration of small molecules, ensuring the maintenance of internal osmolarity [37,38]. While the exact mechanisms connecting these 3 different types of concentration bounds are not currently understood and still require further investigation, a recent theoretical study indicates that large and small molecules jointly interfere with intracellular diffusion and the Gibbs free energies of reactions, resulting in an optimal combined mass density: At lower concentrations, enzymes are not sufficiently saturated with their substrates, while at higher concentrations, the slow down of diffusion limits the substrate supply [39]. The study’s estimate of the optimal dry mass density was highly consistent with observed values in *E*. *coli* [19]. These results indicate that the overall mass concentration limit considered here can be seen as a “coarse-grained” constraint approximating more fundamental physical mechanisms.

The optimal use of dry mass density is also to be expected if we look at the problem from a different, simpler angle: Between 2 cells with all reactions running at the exact same rates, the cell maintaining such rates at a smaller dry mass density will grow faster, since it can reproduce its own biomass in less time (see **Text A in S1 File** for more details). As growth rate is an important determinant of fitness in fast-growing microbes such as *E*. *coli* [40], the resulting selection pressure toward minimal dry mass would continue until eventually other costs, such as the costs of increasingly detailed gene regulation systems, prevent further fine-tuning of the enzyme–substrate relationship.

We wish to emphasize that our conclusions do not rest on the details of these theoretical considerations, but on the quantitative agreement between our predictions and the observed enzyme–substrate relationships in *E*. *coli*. We are not aware of the existence of plausible alternative models that could make equally accurate predictions without fitting any parameters. Accordingly, we conclude that the derivations leading to Eqs (5) and (6) currently provide the best explanation for the observed relationships.

Clearly, other factors than those considered above also affect optimal allocation strategies. For instance, the concentration of membrane-permeable metabolites is often set by external concentrations. Further, the cell might favor higher enzyme levels in order to lower the concentrations of toxic substrates such as reactive oxygen species, weak acids, or formaldehyde. Our analysis in its current form also does not consider posttranslational regulation, such as the suppression of enzyme activities by allosteric regulation or protein modifications. Such regulation does occur for a minority of enzymes in *E*. *coli* under some conditions, and, when it does, our results are no longer expected to hold. Posttranslational regulation plays a stronger role in eukaryotes; given the lack of matching, quantitative proteomics and metabolomics data from eukaryotes, an evaluation of the applicability of our theory beyond prokaryotes currently appears infeasible.

Multiple reactions in central carbon metabolism are reversible. Several of these have been found to operate close to thermodynamic equilibrium, where we expect deviations of the enzyme/substrate concentration ratio toward higher values compared to our equations. Here, Eqs (5) and (6) provide lower bounds for the optimal enzyme concentrations; in contrast to effectively irreversible reactions, a quantitative prediction of these values is impossible unless we consider the complete reaction network, as enzyme concentrations are now interdependent with both substrate and product concentrations [30]. However, 70% of all enzymatic reactions in the *E*. *coli* genome-scale metabolic model are labeled as generally irreversible [31], and many other reactions are likely effectively irreversible in certain conditions; together with the results in **Fig 3**, these considerations indicate that our theory is widely—although not universally—applicable.

The metabolomics data used for **Fig 3** cover 4 orders of magnitude, but are biased toward highly abundant molecules involved in high-flux, central pathways; while *E*. *coli* is able to produce over 1,000 metabolites in total, most of these typically occur at low concentrations, such that the total *E*. *coli* metabolome accounts for only about 10% to 20% of dry mass [41,42] compared to the 3.1% for the 43 metabolites assayed by Gerosa and colleagues [12]. While it is conceivable that the observed relationships only apply to more abundant metabolites and their consuming enzymes, **Fig 3** does not indicate a qualitatively different behavior for metabolites at low mass concentrations. A thorough, genome-wide analysis of the applicability and limits of our theory will have to await the generation of quantitative concentration data for the complete *E*. *coli* metabolome.

In sum, our results highlight the trade-off between the cellular maintenance costs of enzyme and metabolite pools, indicating that their concentrations are approximately balanced toward the parsimonious use of cellular resources. This organizing principle not only improves our understanding of cellular resource allocation, but can also contribute to the optimization of the metabolic efficiency of engineered strains and synthetic cellular systems.

## Materials and methods

### Concentrations

#### Proteins and metabolites

We obtained protein concentrations of *E*. *coli* strain BW25113 for 18 exponential growth conditions on minimal media [15] (S7 Data). For 7 of these conditions, we additionally obtained metabolite concentrations [12] for the same strain (S6 Data).

Individual absolute protein abundances and growth rates for cells growing exponentially in different carbon-limited conditions were obtained from Schmidt and colleagues [15]. Protein mass concentrations (protein mass per cytoplasmic volume) were obtained by first converting the reported absolute protein abundances into protein mass fractions (gram of proteins per total protein mass) by multiplying protein abundances by the molecular weight and normalizing them so that they sum to 1. The resulting fractions were converted to protein mass per dry weight by multiplying them by the ratio of total protein mass to dry mass, *M*_*P*_/*M*_*DW*_. For carbon-limited cells, experimental data from Basan and colleagues [27] can be well described by a linear function of the growth rate *λ*, *M*_*P*_/*M*_*DW*_ = 0.8053−λ×(0.1461 *h*). Finally, the resulting dry weight fractions were divided by the ratio of cytoplasmic volume and dry mass [43], 2.23 μL/mg_DW_ to obtain protein mass per cytoplasmic volume. Metabolite concentrations were obtained from Gerosa and colleagues [12] in units of μmol/gCDW and converted to μmol/μL using the same conversion factor 2.23 μL/mg_DW_ used for the proteins.

#### Enzyme–substrate dissociation constants

For **Fig 3A**, we collected a nonredundant set of enzyme dissociation (Michaelis) constants *K*_m_ of wild-type enzymes from EcoCyc [31], BRENDA [32], and UniProt [33] (S8 Data). All experimental values are from *E*. *coli*, with the exception of 2 metabolite–enzyme pairs where only data from other organisms are available: D-ribulose 5-phosphate–ribose-5-phosphate isomerase A (Ru5P–rpiA) and 1,3-bisphospho-D-glycerate–phosphoglycerate kinase (13DGP–pgk). If more than one *K*_*m*_ was listed across the databases, we first checked if these values were mostly within the same order of magnitude (i.e., if the geometric standard deviation was ≤10); in this case, we used the geometric mean of all available values. Otherwise, we considered the available data for *K*_*m*_ to be too unreliable to be included. For **Fig 1A,** we obtained *K*_*m*_ values from the dataset in reference [11], filtered for the organism *E*. *coli* and restricted to values for reaction substrates rather than products. Metabolite molecular weights were obtained from EcoCyc [31].

### Dominant enzymes

If the unsaturated mass concentration *m*_*E*_[*E*_*free*_]* of enzyme *i* accounts for more than half of the total protein mass utilizing a given substrate *S*, Eq (5) approximately describes the relationship between enzyme and substrate concentration also in the general case (Eq (6)). In this case, we call *E*_*i*_ the “dominant” enzyme for *S*. For an automated identification of dominant enzymes, we used the sybilSBML [44] package in R [45], with the EcoCyc [31] metabolic model for *E*. *coli* exported as an SBML file using Pathway Tools 19.5 [46]. For each metabolite measured in reference [12], we first identified all reactions using it as a substrate according to the metabolic model. The gene-reaction associations given in the EcoCyc model through b-numbers were used to map the reactions to the proteins measured in reference [15].

For each substrate assayed in by Gerosa and colleagues [12], we determined a dominance score (hereafter referred to simply as “dominance”) for each enzyme consuming it and assayed in by Schmidt and colleagues. The dominance of an enzyme was defined as the fraction it contributes to the total mass concentration of all assayed enzymes using the substrate. An enzyme was considered “dominant” for the substrate if its dominance was >0.5, i.e., its molecules constituted more than half of the total protein mass consuming the substrate. We only attempted to assess dominance if more than half of the enzymes capable of consuming a given substrate were assayed in reference [15].

For enzymes with dominance > 0.5, we still did not consider it dominant for further analysis if

- its substrate has a major role besides the involvement with the assigned metabolic enzymes in the EcoCyc model. That is the case for 2 metabolites with major role in gene regulation: Cyclic AMP (cAMP) regulates transcription through varying concentrations of cAMP-CPR, and 2-dehydro-3-deoxy-D-gluconate 6-phosphate is a component of the YebK-2-dehydro-3-deoxy-D-gluconate 6-phosphate transcriptional regulator; accordingly, the metabolic enzymes using these metabolites as substrates are not expected to have a major impact on their concentrations.
- its associated metabolite is in fact a product, not a substrate of the respective reaction. We inferred this by (a) accessing the available condition-dependent reaction directions also measured in Gerosa and colleagues [12]; and (b) for 3 amino acids (L-tyrosine, L-arginine, and Adenine), their respective most dominant enzymes (aspC, argH, and deoD) are in fact catalyzing reactions in their biosynthesis pathways [31].

Dominant enzyme information including their genes, bnumbers, dominance, reversibility, and concentrations are included in **S2 Data**. This file also includes the corresponding information for the second most dominant enzyme in each case.

### Derivations

Let us first consider the simple case of a substrate used by a single irreversible reaction. For an irreversible enzymatic reaction that converts a single substrate into a product according to a general kinetic function *k* ≡ *k*([*S*], *K*_m_, *k*_cat_), the reaction rate is

v=[E]k (7)

with enzyme molar concentration [E] and substrate molar concentration [S]. For irreversible Michaelis–Menten kinetics,

$$k=k_{cat}\frac{\left[S\right]}{[S]+K_{m}},$$ (8)

where *k*_cat_ is the turnover number and *K*_m_ is the enzyme–substrate dissociation (Michaelis) constant. The enzyme and substrate concentrations of this reaction together account for a total mass concentration *M*, measured per volume of the corresponding cellular compartment, e.g., the cytosol; *M* is a linear function of the molar concentrations [*E*] and [*S*], each multiplied with the respective molecular weights (*m*_*E*_ and *m*_*S*_, respectively):

$$M=m_{E}[E]+m_{S}[S]$$ (9)

Maximizing the flux at a given total mass concentration *M* is mathematically equivalent to minimizing *M* at a constant flux; we here consider the latter scenario, assuming that the cell is in a steady state that demands a fixed reaction rate *v*>0. Rearranging Eq (7), we can express [*E*] as a function of *v* and the kinetic function *k*([*S*], *K*_m_, *k*_cat_),

$$[E]=\frac{v}{k}$$ (10)

We assume *v*>0 and thus [*S*]>0 and *k*>0 throughout our derivations. Substituting Eq (10) into Eq (9), we can express the reaction’s total mass concentration, *M*, as a function of the substrate concentration [*S*] and the constants *v*, *K*_m_, *k*_cat_:

$$M=m_{E}\frac{v}{k}+m_{S}[S]$$ (11)

If *M* is minimal, a necessary condition is that the derivative of Eq (11) with respect to [*S*] must be zero (at constant *v*):

$${\frac{dM}{d[S]}|}_{[S]={\left[S\right]}^{*}}=0$$ (12)

We thus have

$$-m_{E}\frac{v^{*}}{{\left(k^{*}\right)}^{2}}\frac{dk}{d[S]}+m_{S}=0$$ (13)

We can simplify the further derivation if we divide all terms in Eq (13) by *m*_*S*_ and consider the ratio *a* ≔ *m*_*E*_/*m*_*S*_:

$$a\frac{v^{*}}{{\left(k^{*}\right)}^{2}}\frac{dk}{d[S]}=1$$ (14)

Substituting the flux *v* using Eq (7):

$$a\frac{{\left[E\right]}^{*}}{k^{*}}\frac{dk}{d[S]}=1$$ (15)

To calculate the derivative, we assume irreversible Michaelis–Menten kinetics; however, the derivation can proceed identically for any other irreversible kinetic rate law.

For irreversible Michaelis–Menten kinetics (Eq (8)), Eqs (14) and (15) result, respectively, in

$$v^{*}=k_{cat}\frac{({{\left[S\right]}^{*})}^{2}}{aK_{m}}$$ (16)

$$a{\left[E\right]}^{*}={\left[S\right]}^{*}\left(1+\frac{{\left[S\right]}^{*}}{K_{m}}\right)$$ (17)

We note that Eq (17) does not depend on *k*_cat_. Combining Eq (17) with Eq (3) of the main text results in the equality between the mass concentration of substrate and free enzyme,

$$m_{S}{\left[S\right]}^{*}=m_{E}{\left[E_{free}\right]}^{*}$$ (18)

Both Eq (16) and (17) can further be solved for [*S*]* to give, respectively,

$${\left[S\right]}^{*}=\sqrt{\frac{aK_{m}v^{*}}{k_{cat}}}$$ (19)

$${\left[S\right]}^{*}=\frac{K_{m}}{2}\left(\sqrt{1+\frac{4a{\left[E\right]}^{*}}{K_{m}}}-1\right)$$ (20)

Substituting Eq (19) in Eq (16) and Eq (20) in Eq (17), we have, respectively,

$$v^{*}=k_{cat}\left({\left[E\right]}^{*}-\frac{{\left[S\right]}^{*}}{a}\right)$$ (21)

$${\left[E\right]}^{*}=\frac{v^{*}}{k_{cat}}+\sqrt{\frac{K_{m}v^{*}}{ak_{cat}}}$$ (22)

Here, [*S*]* is given by Eq (20). In both equations, we note that the second term on the right-hand side is a consequence of the incomplete enzyme saturation by the metabolite.

#### Error in predicted substrate concentration due to uncertainties in ***K***_**m**_

Consider the mass concentrations (densities) at optimality of enzyme, $c_{E}^{*}=m_{E}{\left[E\right]}^{*}$, and substrate, $c_{S}^{*}=m_{S}{\left[S\right]}^{*}$. According to Eq (5),

$$c_{E}^{*}=c_{S}^{*}\left(1+\frac{c_{S}^{*}.}{{\tilde{K}}_{m}}\right)=c_{S}^{*}+\frac{{c_{S}^{*}}^{2}}{{\tilde{K}}_{m}}$$

$$\Rightarrow c_{S}^{*}=\frac{{\tilde{K}}_{m}}{2}\left(\sqrt{1+\frac{4c_{E}^{*}}{{\tilde{K}}_{m}}}-1\right)$$

$$\Rightarrow \frac{\partial c_{S}^{*}}{\partial {\tilde{K}}_{m}}=\frac{c_{S}^{*}}{{\tilde{K}}_{m}}\left(1-\frac{1+\frac{c_{S}^{*}}{{\tilde{K}}_{m}}}{\sqrt{1+\frac{4c_{E}^{*}}{{\tilde{K}}_{m}}}}\right)\leq \frac{c_{S}^{*}}{{\tilde{K}}_{m}}$$

$$\Rightarrow \frac{\Delta c_{S}^{*}}{c_{S}^{*}}\leq \frac{\Delta {\tilde{K}}_{m}}{{\tilde{K}}_{m}},$$ (23)

where the second to last inequality follows from the fact that the partial derivative is known to be positive, and the last line follows from the law of error propagation. As $\Delta c_{S}^{*},\;c_{S}^{*},\;{\Delta \tilde{K}}_{m}$, and ${\tilde{K}}_{m}$ are all scaled by the same molar masses relative to Δ[*S*]*, [*S*]*, Δ*K*_m_, and *K*_m_, respectively, it follows that the relative error in [*S*]* is at most that of *K*_m_.

#### Optimality at the systems level

Enzymatic reactions in biological cells are not isolated: The same substrate is often consumed by multiple enzymes, and the same enzyme may utilize multiple substrates. We thus need to generalize the above derivation to the systems level, considering all metabolic reactions within one cellular compartment (e.g., the cytosol) simultaneously.

A nonzero rate *v*_*j*_ of reaction *j* can then be described using any reaction kinetics as

$$v_{j}={[E}_{j}]k_{j},$$ (24)

where the effective rate per enzyme $k_{j}=k_{j}\left([S_{i}],k_{{cat}_{j}},K_{m_{ij}}\right)$ is a function of the metabolite concentrations [*S*_*i*_] and respective turnover number $k_{{cat}_{j}}$, and Michaelis constants $K_{m_{ij}}$ (in the further derivations, we assume $K_{m_{ij}}=0$ if the metabolite *i* is not involved in the reaction *j*). We assume that the cell is in a given metabolic state, i. e., all reactions have a fixed rate *v*_*j*_ ($\vec{v}$ = const). Below, we are only concerned with active reactions (*v*_*j*_>0), and we thus drop metabolites and enzymes involved only in nonactive reactions from further consideration (i.e., we assume [*S*_*i*_]>0 and [*E*_*j*_]>0 for all *i* and *j* without loss of generality).

In this metabolic state, the metabolism of a given cellular compartment accounts for a total mass concentration *M*_*total*_; this can be calculated as the sum of all enzyme and metabolite molar concentrations, each term multiplied by the corresponding molecular weight:

$$M_{total}=\sum_{j}m_{E_{j}}{[E}_{j}]+\sum_{i}m_{S_{i}}[S_{i}]$$ (25)

The derivation proceeds largely as above. We can rearrange Eq (25) to express each enzyme concentration [*E*_*j*_] as a function of *v*_*j*_ and the vector of effective rates (which itself is a function of metabolite concentrations [*S*_*i*_]) as

$${[E}_{j}]=\frac{v_{j}}{k_{j}}$$ (26)

It follows that for any vector of reaction rates $\vec{v}$ and any vector of nonzero metabolite concentrations [*S*_*i*_], there always exists a matching vector of enzyme concentrations [*E*_*j*_]. Substituting Eq (26) into Eq (25), we obtain

$$M_{total}=\sum_{j}m_{E_{j}}\frac{v_{j}}{k_{j}}+\sum_{i}m_{S_{i}}{[S}_{i}],$$ (27)

which is now only a function of metabolite concentrations [*S*_*i*_], kinetic parameters and the constants $\vec{v},{\vec{m}}_{E},{\vec{m}}_{M}$.

If this metabolic state has the lowest possible mass concentration (i.e., *M*_*total*_ is minimal with respect to all metabolite concentrations), then all partial derivatives must vanish,

$$0={\frac{\partial M_{total}}{\partial {[S}_{l}]}|}_{[S_{l}]={\left[S_{l}\right]}^{*}}=-\sum_{j}m_{E_{j}}\frac{v_{j}^{*}}{{\left(k_{j}^{*}\right)}^{2}}\frac{\partial k_{j}}{\partial {[S}_{l}]}+m_{S_{l}},$$ (28)

for all metabolites *l* (we keep the index *i* reserved for the sum of metabolites and use *l* for the respective partial derivatives, in order to avoid confusion in later equations). Dividing all terms in Eq (28) by $m_{S_{l}}$ and rearranging, we obtain

$$\sum_{j}\frac{a_{lj}v_{j}^{*}}{{\left(k_{j}^{*}\right)}^{2}}\frac{\partial k_{j}}{\partial {[S}_{l}]}=1,$$ (29)

where $a_{lj}≔m_{E_{j}}/m_{S_{l}}$ is the ratio of the molecular weights of enzyme *E*_*j*_ and its substrate *S*_*l*_. Using Eq (24) to resubstitute the reaction rates *v*_*j*_ into Eq (29) leads to

$$\sum_{j}\frac{a_{lj}{\left[E_{j}\right]}^{*}}{{\left(k_{j}^{*}\right)}^{2}}\frac{\partial k_{j}}{\partial [S_{l}]}=1$$ (30)

This equation can be solved for arbitrary kinetic functions (for any explicit dependency of *k*_*j*_(***S***) on the metabolite concentrations ***S***), provided these are effectively irreversible.

If all reactions *j* follow generalized irreversible Michaelis–Menten kinetics of the “convenience kinetics” form[47],

$$k_{j}=k_{cat_{j}}\prod_{i}\left(\frac{\left[S_{i}\right]}{[S_{i}]+K_{m_{ij}}}\right),$$ (31)

where the kinetic parameters consist of turnover numbers $k_{{cat}_{j}}$ and Michaelis constants $K_{m_{ij}}$, then Eq (30) results in

$$\sum_{j}\frac{a_{lj}{\left[E_{j}\right]}^{*}}{{\left[S_{l}\right]}^{*}(1+\frac{{\left[S_{l}\right]}^{*}}{K_{m_{lj}}})}=1,$$ (32)

which only depends on the concentration and Michaelis constants of a single substrate *S*_*l*_ and is independent of turnover numbers $k_{{cat}_{j}}$. Thus, the contribution of each individual metabolite to the total cellular cost in a maximally efficient metabolic system can be considered in isolation. Also considering irreversible (generalized Michaelis–Menten) convenience kinetics, Eq (29) results in

$$\sum_{j}\frac{a_{lj}v_{j}^{*}K_{m_{lj}}\varphi_{lj}^{*}}{k_{{cat}_{j}}}={{\left([S_{l}\right]}^{*})}^{2},$$ (33)

where

$$\varphi_{lj}^{*}≔\prod_{l^{\prime }\neq l}\left(\frac{K_{m_{l^{\prime }j}}}{{\left[S_{l^{\prime }}\right]}^{*}}+1\right)$$ (34)

is the contribution of the other metabolites *l*′≠*l* used as substrates in reaction *j*.

Combining Eq (32) with Eq (3) directly generalizes Eq (18), now considering the concentration of all free enzymes *j* using a substrate *l*:

$$m_{S}{\left[S_{l}\right]}^{*}=\sum_{j}m_{E_{j}}{\left[E_{j,free}\right]}^{*},$$ (35)

where [*E*_*j*,free_] is the concentration of the fraction of enzyme *E*_*j*_ not bound to its substrate *S*_*l*_.

This equation applies to a complete metabolic system of effectively irreversible reactions following generalized Michaelis–Menten kinetics: The optimally cost-efficient concentration of each metabolite [*S*_*l*_] in a given metabolic state (i.e., at given reaction rates $\vec{v}$) depends only on the concentrations of the enzymes consuming it, their affinities $K_{m_{lj}}$ for the metabolite, and the enzyme/metabolite molecular weight ratios *a*_*lj*_, but is independent of turnover numbers and reaction rates.

If one of the summands in Eq (35) is close to 1, it will dominate this expression, and we approximately recover Eq (5) of the main text. The dominant term will usually correspond to the enzyme with the highest *a*_*lj*_*E*_*j*_; this is what is shown in **Fig 3A** of the main text.

## Supporting information

S1 File File containing Supporting information Text A and Supporting information Figs A, B, and C. (PDF)

S2 File Zip archive containing supporting code. (ZIP)

S1 Data Data related to Fig 1. (XLSX)

S2 Data Dominant enzyme/substrate abbreviations and data related to Fig 3. (XLSX)

S3 Data Data related to Fig A in S1 File. (XLSX)

S4 Data Data related to Fig B in S1 File. (XLSX)

S5 Data Data related to Fig C in S1 File. (XLSX)

S6 Data Metabolome data from Gerosa and colleagues [12]. (CSV)

S7 Data Proteome data from Schmidt and colleagues [15]. (CSV)

S8 Data *K*_m_ data. (CSV)

## Abbreviations

13DGP–pgk: 1,3-bisphospho-D-glycerate–phosphoglycerate kinase
cAMP: cyclic AMP
GAL1P: alpha-D-galactose 1-phosphate
GaLT: galactose-1-phosphate uridylyltransferase
GMFE: geometric mean fold error
Ru5P–rpiA: D-ribulose 5-phosphate–ribose-5-phosphate isomerase A
