# Supplementary material for: On the optimality of the enzyme–substrate relationship in bacteria
Source: PLoS Biol. 2021 Oct 26;19(10):e3001416. doi: 10.1371/journal.pbio.3001416 (PMC8547704; doi:10.1371/journal.pbio.3001416)
Supplement: S1 File — (PDF) [file pbio.3001416.s001.pdf]

Supporting Information:

On the optimality of the enzyme-substrate relationship in bacteria

Hugo Dourado, Matteo Mori, Terence Hwa, Martin J. Lercher

Contents

Text A: Global mass concentration and the optimality of growth rate and dry mass density **2**

Supporting Figures **4**

Supporting References **7**

Text A: Global mass concentration and the optimality of growth rate and dry mass density

Let's define the (column) biomass vector $\boldsymbol{b} = ([S_i], [P])^T$ as the combination of the individual metabolite molar concentrations $[\boldsymbol{S}]$ (a vector) and the total protein molar concentration $[P]$ (a scalar). The *molar* conservation of each of these components at steady-state cellular growth with reaction rates $\boldsymbol{v}$ is conveniently expressed in matrix notation as

$$N\boldsymbol{v} = \lambda \boldsymbol{b} \ ,$$

where N is the stoichiometric matrix of internal components, including a protein production reaction [1]. For each biomass component, this equation describes its "local" balance in units of moles between production, consumption, and dilution by growth at rate λ. In addition, we can consider a global *mass* balance at steady-state, which arises as a weighted sum of all equations of local mass balance. This corresponds to multiplying the last equation from the left by the row vector $\boldsymbol{m}^T$ of molecular masses (including an average molecular mass of proteins m_P),

$$\boldsymbol{m}^T N\boldsymbol{v} = \lambda \boldsymbol{m}^T \boldsymbol{b} = \lambda M_{total} \ ,$$

where the last equality comes from the sum of all mass concentrations, as in equation (4) of the main text. Let's note that mass conservation of chemical reactions also implies that each component in the row vector ($\boldsymbol{m}^T N$) is non-zero only for reactions in the cell boundary, i.e., for reactions transporting mass into or out of the cell [2], so that the left-hand side of the last equation depends effectively only on those boundary reactions. For the convenience of the following discussion, let's identify the left-hand side of the last equation as the biomass production rate v_{bio} , i.e., is the global net difference between the mass flux into and out of the cell, which has to accumulate in the form of biomass,

$$v_{bio} := \boldsymbol{m}^T N\boldsymbol{v} \quad .$$

From the last definition, we restate the global mass balance at steady-state growth simply as

$$v_{bio} = \lambda M_{total} \quad .$$

Let's now consider the kinetic relationship between each enzyme concentration with its reaction rate and reactants $[E_j] = v_j/k_j$, for some given kinetic function $k_j([\boldsymbol{S}])$ (equation (24)), so each protein concentration $[E_j]$ in our model is uniquely determined by the respective flux v_j and substrate concentrations $[\boldsymbol{S}]$. We can thus eliminate the protein concentrations from the global mass balance equation, resulting in a relationship between v_{bio}, λ, and M_{total} that depends exclusively on reaction rates $\boldsymbol{v}$ and metabolite concentrations $[\boldsymbol{S}]$:

$$v_{bio}(\boldsymbol{v}) = \lambda M_{total}(\boldsymbol{v}, [\boldsymbol{S}]) \quad ;$$

Here, $M_{total}(\boldsymbol{v}, [\boldsymbol{S}])$ is determined by the sum in equation (4) of the main text. The important point to note now is that for any given distribution of fluxes $\boldsymbol{v}$, v_{bio} becomes fixed; according to the last equation, the distribution of substrate concentrations $[\boldsymbol{S}]^*$ that results in maximal growth rate ($\lambda = \lambda^{max}$) is then the same that results in minimal dry mass density M_{total}^{min} while maintaining the fluxes $\boldsymbol{v}$**.** Thus, the simple optimization of dry mass density presented here can be understood as an approximation to the more general problem of the growth rate optimization of self-replicators, which additionally account for the "local" mass conservation of each cellular component and the reaction network structure [1,2].

Supporting Figures

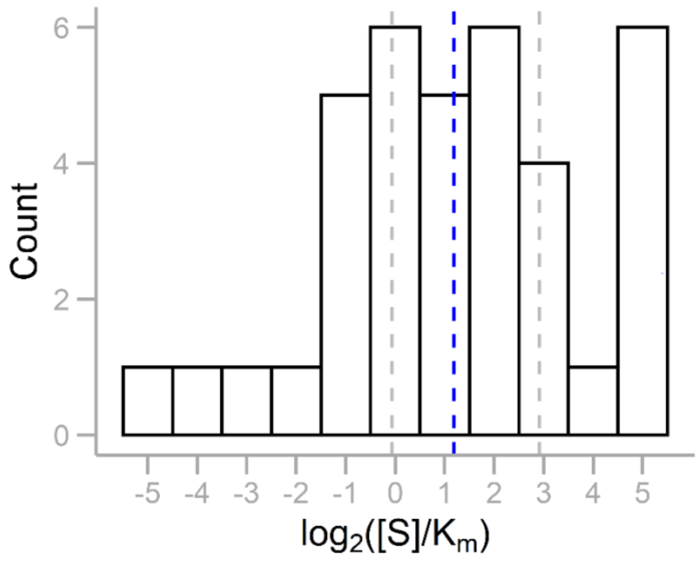

Fig A. The ratio between absolute metabolite concentration in the glucose reference condition[3] and the geometric mean across all known *E. coli* K_m values for this substrate is distributed around 2. Median: 1.96 (dashed blue line), 25%-quantile=0.96, 75%-quantile=7.53 (dashed grey lines); *N*=37, as no K_m values are available for 6 of the 43 metabolites assayed in Ref.[3]. The underlying data can be found in **S3 Data**.

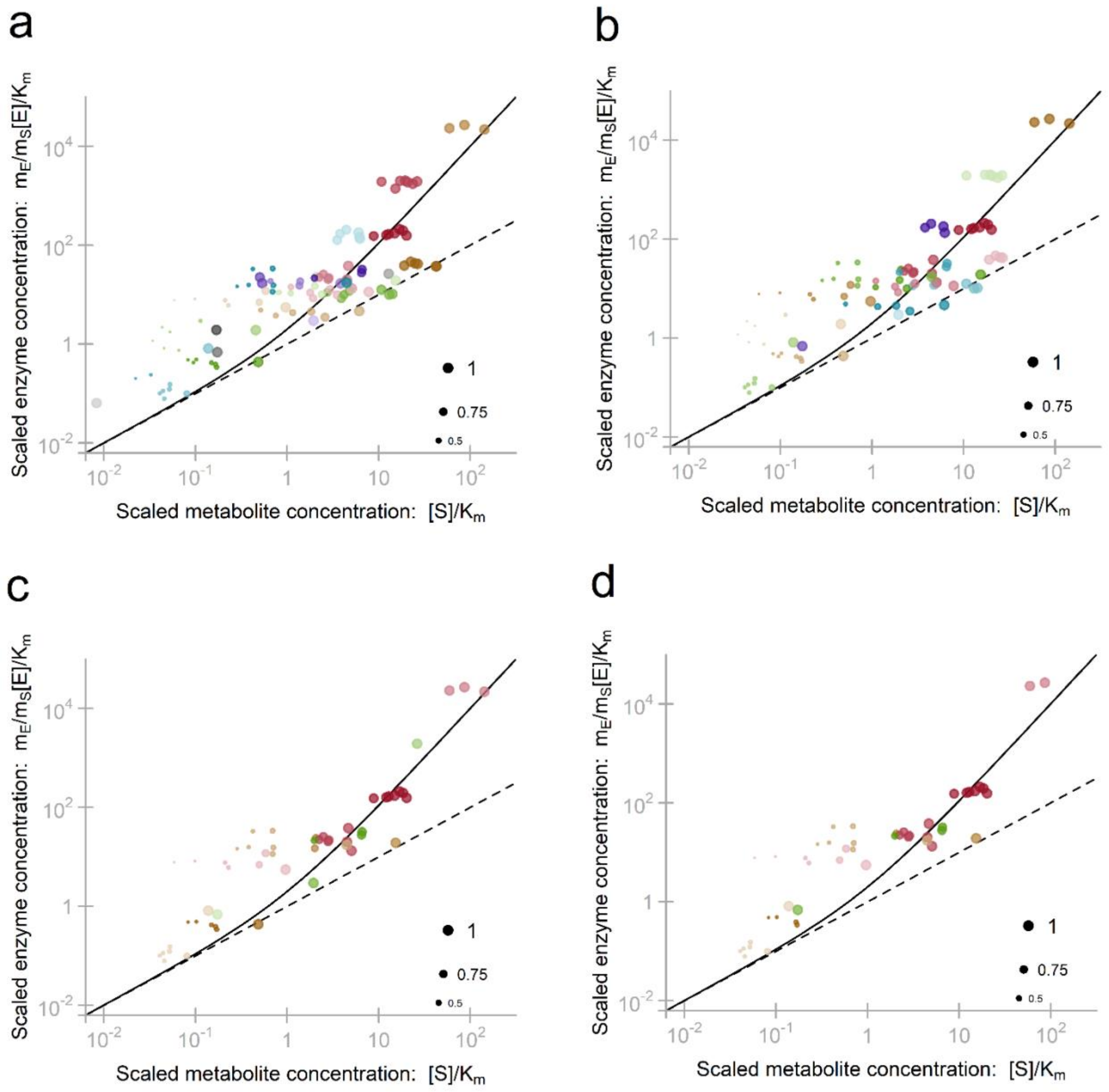

Fig B. Predictions of substrate concentrations from enzyme concentrations become more accurate for more restrictive definitions of dominance. Same as Fig. 3a of the main text, but including enzyme-metabolite pairs where the enzyme accounts for different minimal fractions of the total enzyme mass capable of consuming the metabolite (different dominance cutoffs; Fig. 3a shows dominance ≥ 0.5): **(a)** dominance ≥ 0.4 (N=113, r^2=0.44, GMFE=2.90), **(b)** dominance ≥ 0.45 (N=92 r^2=0.49, GMFE=2.78), **(c)** dominance ≥ 0.55 (N=53, r^2=0.60, GMFE=2.55), and **(d)** dominance ≥ 0.6 (N=47, r^2=0.61, GMFE=2.72). The underlying data can be found in **S4 Data**.

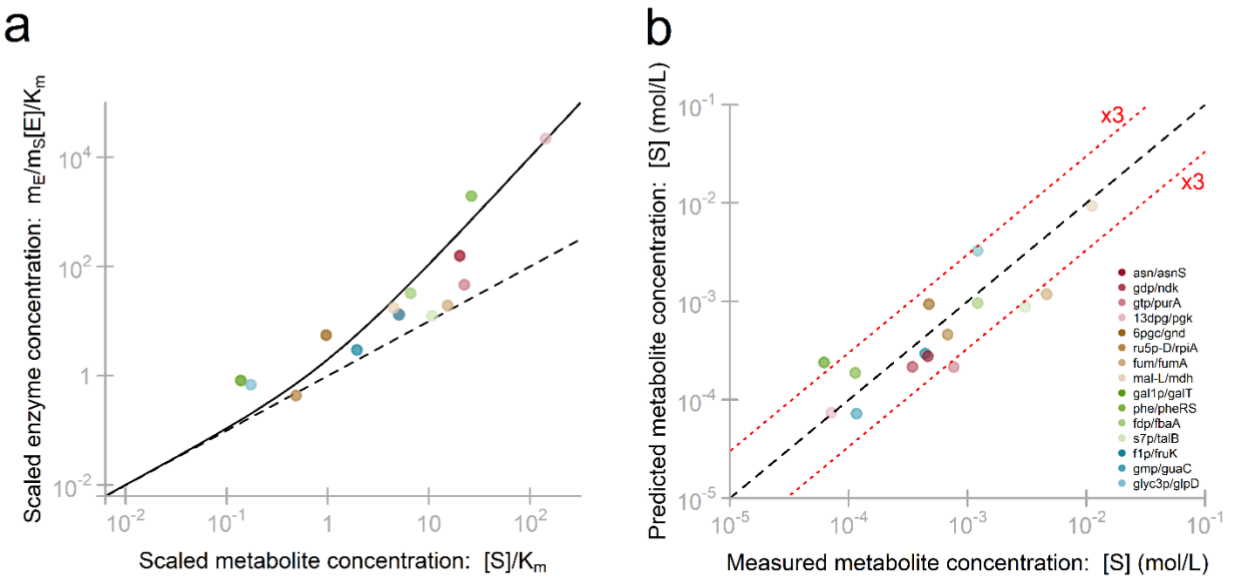

Fig C. Predictions of substrate concentrations from enzyme concentrations are more accurate when considering only the highest saturation for each enzyme-substrate pair. (a) Comparison of predicted (solid line) and experimentally observed (dots) mass concentration combinations of dominant enzymes and their substrates. **(b)** Comparison of predicted (y-axis) and experimentally observed (x-axis) molar metabolite concentrations. Data points are color-coded by reaction. Same as Fig. 3a,b of the main text, but showing only the data point with the highest saturation for each enzyme-substrate pair (N = 15, r^2= 0.72, GMFE = 1.96). The underlying data can be found in **S5 Data**.

Supporting References

1. Molenaar D, van Berlo R, de Ridder D, Teusink B. Shifts in growth strategies reflect tradeoffs in cellular economics. Mol Syst Biol. 2009;5: 323. doi:10.1038/msb.2009.82
2. Dourado H, Lercher MJ. An analytical theory of balanced cellular growth. Nat Commun. 2020;11: 1226. doi:10.1038/s41467-020-14751-w
